# Health impacts of climate change and role of the health sector in mitigating carbon emissions

**DOI:** 10.1007/s00120-025-02698-z

**Published:** 2025-11-12

**Authors:** E. Rothwell, Jonny Groome

**Affiliations:** 1 University Hospitals Bristol and Weston NHS Trust, Bristol, UK; 2 Barts Health NHS Trust, London, UK; 3 Nuffield Health, Epsom, UK; 4 University College London, London, UK; 5 Paediatric Anaesthetic Department Office, Level 4, The Royal London Hospital, Whitechapel Road, E11FR London, UK

**Keywords:** Climate crisis, Planetary health, Sustainability, Sustainable hospitals, Climate mitigation, Klimakrise, Planetare Gesundheit, Nachhaltigkeit, Nachhaltige Krankenhäuser, Klimaschutz

## Abstract

The climate crisis has been identified as the largest threat to human health; paradoxically, the healthcare sector is responsible for 5% of the global greenhouse gas emissions that are driving this crisis. These emissions are largely due to carbon-intensive facilities, energy use, complex global supply chains, transportation and pharmaceuticals. In its role of safeguarding the health of both current and future populations, the healthcare sector must take actions to minimise its environmental impact. Strategies for emission reduction include sustainable infrastructure, clinical practice innovations, and procurement and supply chain reform. This article aims to examine current evidence on the health impacts of climate change and explore strategies through which the healthcare sector can reduce its environmental impact while continuing to deliver high-quality care.

## Abstract (de)

Die Klimakrise gilt als größte Bedrohung für die menschliche Gesundheit, und paradoxerweise ist der Gesundheitssektor für 5 % der weltweiten Treibhausgasemissionen verantwortlich, die diese Krise befeuern. Die Emissionen sind größtenteils bedingt durch Einrichtungen mit hohem Treibhausgasausstoß, durch Energieverbrauch, komplexe globale Lieferketten, Transport und Arzneimittel. Angesichts seiner Funktion als Garant der Gesundheit heutiger wie auch kommender Generationen muss der Gesundheitssektor Maßnahmen ergreifen, um seine negativen Auswirkungen auf die Umwelt zu minimieren. Zu den emissionsreduzierenden Strategien zählen eine nachhaltige Infrastruktur, Innovationen in der klinischen Praxis sowie Verbesserungen in Bezug auf Beschaffung und Lieferketten. Im vorliegenden Beitrag wird die aktuelle Evidenz zu Auswirkungen des Klimawandels auf die Gesundheit beleuchtet. Zudem werden Strategien betrachtet, mit deren Hilfe der Gesundheitssektor seine Umweltfolgen reduzieren und zugleich eine qualitativ hochwertige Versorgung aufrechterhalten kann.

## Introduction

Climate change has been identified as the largest threat to human health [1]. The intersection between climate change and public health is clear, as anthropogenic greenhouse gas (GHG) emissions alter environmental conditions and consequently impact human health. The health sector is paradoxically a significant contributor to GHG emissions—it is responsible for approximately 5% of global emissions [2]. Given its mission of safeguarding health, the sector has an ethical obligation to minimise its environmental impact. This article aims to examine the health impacts of climate change and highlight the role of the health sector in reducing these emissions while maintaining or improving patient care.

## Impacts of climate change on health

### Direct effects

Climate change is contributing to a rise in extreme weather events such as heatwaves, droughts, extreme precipitation and wildfires [1]. In 2023 infants and adults over 65 experienced a record high number of heatwave days—an average of 13.8 per person, up from 4.7 heatwave days in the period 1886–2005 [1]. During heatwaves, there is an increase in all-cause mortality—the leading cause of death is cardiovascular disease [3]. Mortality is higher for those with underlying vulnerabilities, such as chronic disease, and among those at the extremes of age [3]. Analysis of the heatwave that Europe experienced between 23 June and 2 July 2025 estimated a total of 2305 excess deaths due to heat across 12 major Mediterranean cities, with 60% of these attributable to anthropogenic climate change [4]. Heatwaves also affect the health of our economy, with an estimated 512 billion potential work hours lost in 2023 [1].

In 2017, Hurricane Maria hit Puerto Rico, resulting in a 60% increase in mortality in the 3 months following the storm [5]. One emergency department found that 10% of attendances for injury were hurricane-related [6]. In addition to the direct effect of injury, damage to key infrastructure affected the health of the population for years thereafter—as evidenced by increased mortality risk from diseases such as cardiovascular disease and Alzheimer’s disease [7].

### Indirect effects

Heatwaves exert additional risks to health as they increase a population’s risk of food insecurity [8]. This is largely through reduced crop yields and labour capacity, supply chain disruption and reduced access to water and sanitation [8]. Globalisation means that these impacts are felt across supply chains; however, subsistence farmers and indigenous peoples are most vulnerable. Indigenous children experience higher levels of malnutrition compared to non-indigenous children [9] and are therefore most vulnerable to food shortages.

Rapid climate changes are accompanied by a rapidly rising level of climate migration

Vector-borne diseases are climate sensitive, as the ability of the vector to survive depends on the climate. Dengue is an example of a climate-sensitive disease whose incidence is already increasing. The global burden of dengue has increased sharply over the past three decades, with over 7.6 million cases being reported in 2024 [10]. Transmission is via the *Aedes* mosquito, and the average transmission risk (R0, basic reproduction number) has increased for both main vectors—*Aedes albopictus* (46.3%) and *Aedes aegypti* (10.7%)—between the periods 1951–1960 and 2014–2023, with the greatest increase seen in countries with a high Human Development Index (HDI; [1]).

Throughout history, ecological change has shaped migration. The rapidly changing climate has been accompanied by a rapidly increasing rate of climate migration. The Intergovernmental Panel on Climate Change (IPCC) projects that over 1 billion people globally could be exposed to coastal-specific climate hazards by 2050, forcing potentially 10–100 million people to leave their homes [11]. A systematic review of the health effects of climate migration highlights poorer mental health, food and water insecurity, lack of housing and sanitation and marginalisation as key issues [12]. Additionally, migration from rural to sub-urban/urban areas is set to increase from 55% in 2018 to 68% by 2050 [13]. This is associated with specifically increased health risks from cancer, hypertension, cardiovascular disease and type 2 diabetes [12].

If effectively planned and executed, climate migration could reduce migrant populations’ climate vulnerability while avoiding the risks to health cited above. Boston Consulting Group have estimated there will be a 7-million-person worker shortage in the Green Energy Transition by 2030 [14]. A global framework for climate migration, creating safe and legal routes for workers to enter such jobs, could accelerate the energy transition and reduce some of the vulnerabilities that migrating communities experience, such as social isolation and economic disadvantage.

Indirect effects on health can be far reaching. Women experiencing climate-related disasters have been shown to be at increased risk of interpersonal violence—those who reported exposure to more than four disasters had eight times the odds [15].

## Climate change and health inequalities

The health impacts of climate change are not distributed equally. Socioeconomically disadvantaged groups, indigenous communities, older populations, children and those with pre-existing health conditions are especially vulnerable. Structural inequalities such as racism, limited access to healthcare and environmental exposures exacerbate these vulnerabilities. In addition, decarbonisation interventions, if not designed and evaluated with these factors in mind, have the potential to further exacerbate inequalities. In this way, the climate crisis can be seen as a public health threat that must be addressed systematically.

## Carbon footprint of the health sector

The health sector accounts for approximately 5% of GHG emissions globally [2]. Within the sector, emissions can be categorized as follows: Scope 1—from directly owned/controlled sources; Scope 2—indirect emissions from purchased energy; Scope 3—all other indirect emissions from goods, services and supply chains [16]. Carbon footprinting exercises have identified key carbon ‘hotspots’ in healthcare systems—facilities and energy use (10% of the National Health Service [NHS] footprint), supply chain emissions, transportation (22% globally) and medicines/chemicals [2, 16]. These analyses are important in devising strategies to reduce emissions.

## Strategies for emission reduction in healthcare

As significant contributors to anthropogenic climate change, healthcare systems across the world have a role to play in emission reduction and mitigation. To this end, the World Health Organization (WHO) has set up the Alliance for Transformative Action on Climate and Health (ATACH). Of the 94 countries in this alliance, 45 have pledged a net zero commitment [17]. Despite this, health-sector-related GHG emissions continue to rise—up 9.5% in 2021 from 2020, and 36% higher than 2016 [1]. Strategies for emission reduction often go hand in hand with cost savings and improved patient care. Broadly, areas for emission reduction can be classified into (a) sustainable infrastructure, (b) clinical practice innovations and (c) procurement and supply chain reform.

## Sustainable infrastructure

Infrastructure accounts for a significant proportion of the total environmental impact of healthcare. Healthcare Without Harm (HCWH) are an international non-governmental organisation (NGO) working with healthcare providers globally to reduce the sector’s environmental impact. In collaboration with the WHO, they have produced a set of specific actions relating to energy efficiency, green building design, alternative energy generation, transportation, food, waste, and water [18].

### Energy efficiency

Energy inefficiency contributes to hospitals’ GHG emissions and is also associated with significant financial burden. Hospitals are very energy intensive, owing to the continuous nature of their use. Operating theatres have a requirement for strict temperature regulation, air exchange rates and continuous ventilation to maintain sterility, contributing to their energy intensity [19]. Multiple strategies for improving energy efficiency have been identified—conducting energy audits, retrofitting older buildings with more energy-efficient systems, lighting optimisation, smart energy management systems and renewable energy integration. Simple acts such as turning off heating, ventilation and air conditioning (HVAC) systems can lead to energy savings of over € 35,000 per year [20].

### Energy generation

Given the energy intensity of hospitals, switching to 100% renewable energy has potential for huge reductions in the environmental impact of healthcare. The adoption of on-site solar could generate a large proportion of a hospital’s energy requirements [21]. It is estimated that close to 1 billion people in low- and lower–middle-income countries are served by healthcare facilities without reliable electricity access [22]. Decentralised solar energy has the potential to provide energy to these sites, without the delays often associated with a requirement for connection to the grid [22]. Thus, solar energy has huge potential in reducing global health inequalities.

Heat pumps are increasingly being used in both domestic and commercial buildings. A hospital in Sønderborg, Denmark, has installed two heat pumps that use waste heat to deliver efficient heating and cooling; they meet the hospital energy demands and contribute surplus heat to the local heating network [23].

Where on-site projects may be constrained, power purchase agreements make it possible for hospitals to fund renewable energy projects that feed electricity to the grid and then draw energy from the grid at a fixed price. Modelling from the charity Possible has shown that by using community wind projects, which divert profits from the company shareholders back to the community, two NHS Trusts in the United Kingdom could save £ 2.6 million in healthcare costs over 10 years [24].

The increased integration of renewable energy sources has the potential to shelter healthcare facilities from fluctuations in energy costs, provide resilience against power outages and other disruptions, and simultaneously deliver carbon savings.

### Green building design and certification

Green building certification has helped to accelerate adoption of key new technologies in green building design. A widely used framework for sustainable hospital design is Leadership in Energy and Environmental Design (LEED) Certification. This is a set of standards for environmental building design, such as mandating the use of advanced ventilation systems. The adoption of measures outlined by LEED can lead to a predicted 30–50% reduction in total building energy use compared to non-certified buildings [25].

In our changing climate, there is a growing need for hospitals of the future to be built with resilience in mind, with flood-resistant structures, heat-resistant materials and decentralised renewable energy [19].

### Food

The global food system contributes one third of all GHG emissions [26], with a large proportion in the United Kingdom (70%) coming from red meat and dairy alone [1]. Increased consumption of plant-based foods has been shown to be associated with reduced prevalence of diseases such as heart disease, obesity, type 2 diabetes and cancer as well as with a reduction in all-cause mortality [27]. When compared with a diet rich in meat, a vegan diet results in 25.1% lower GHG emissions [28]. Plant-based meals in hospitals reduce the environmental impact of food and have health benefits for patients [1, 27]. In 2022, the organisation Greener By Default worked with 11 New York City hospitals to incorporate plant-forward menus, leading to significant cost savings ($ 500,000 annually) and carbon reductions (1/3 reduction in carbon footprint; [29]).

### Waste

Waste contributes a relatively small proportion of the carbon footprint of healthcare (5% in the NHS [16]); however, it can have far-reaching environmental impacts, owing to the toxic nature of chemicals/materials being disposed [30]. As a system, healthcare should aim to eliminate waste, moving towards a circular economy where products are designed for re-processing and longevity. This requires a major shift in supply chains. There are multiple Europe-wide initiatives aimed at increasing circularity in healthcare systems and reducing the use of single-use plastics [30]. One such project is PVC-Free Healthcare*.* Polyvinyl chloride (PVC) is a major component of medical devices. Its production and disposal are associated with significant environmental and health risks owing to the release of toxic dioxins [31]. The project aims to find alternatives to this toxic, hard-to-recycle material [31].

Certain waste streams in healthcare must be safely disposed of, to avoid risk to human health—for example infectious waste, sharps and toxic pharmaceuticals [30]. Currently, much of this waste is incinerated, producing a wide range of toxic emissions such as CO_2_ and nitrogen oxides [30]. Lower carbon alternatives to incineration include autoclaving, recycling and biodigestion [30]. Healthcare workers also have a key role in reducing the environmental impact of waste by ensuring that only waste that requires this carbon-intensive fate enters these waste streams.

## Clinical practice innovations

### Telemedicine

In the United Kingdom, patient travel accounts for 5% of the NHS carbon footprint [16]. NHS England have estimated that approximately 232 million road miles could be avoided annually by using virtual appointments—a potential saving of 426 t of CO_2_e per year [16]. The challenge is greater in larger, more rural areas. In British Colombia, Canada, one paper estimates an average travel distance of 18.94 km (2.89 kg CO_2_e) per emergency department visit in urban areas and 91.2 km (13.92 kg CO_2_e) in rural areas [32]. Increasing the use of virtual appointments and telehealth could reduce these emissions [32]. However, telemedicine services are not carbon neutral, given the embodied carbon in computing systems and the hosting of online services on servers. It is estimated that where patients are travelling more than 7.2 km, the benefits would be realised [33]. The increasing integration of telemedicine has the potential to exacerbate health inequalities, for example due to different levels of digital literacy; this highlights the importance of intersectional analysis to ensure that the green transition is a just one [34].

### Preventative healthcare

Focusing on the prevention of ill health is inherently sustainable, as it reduces demands and the use of healthcare services. The Getting It Right First Time (GRIFT) programme aims to identify the right *first *intervention for patients, thus reducing the need for revision/re-operations [35]. One emergent theme was that early and intense rehabilitation could decrease the length of stay, post-operative complications and costs—all of which are associated with increased emissions [35]. Pre-operative care focusing on interventions such as detection of anaemia, exercise and pre-operative walking aids has the potential to reduce complications by 30–80% [36], thus reducing emissions and costs.

Kidney care is one of the most resource-intensive specialities in healthcare, especially in the later stages of the disease—where patients require renal replacement therapy [37]. Haemodialysis and peritoneal dialysis involve significant emissions from regular patient and staff travel, large volumes of water and energy usage, and plastic waste [37]. KitNewCare is an EU-funded public–private partnership that aims to generate evidence-based and scalable interventions to improve the quality and sustainability of kidney care. Once identified, more than 450 healthcare providers in over eight European countries will adopt the innovations from KitNewCare [38]. Prevention forms a key part of the decarbonisation model, aiming to increase levels of early diagnosis and effective early management to prevent progression [38].

### Anaesthesia

Anaesthetic gases have long been recognised as having a detrimental effect on global warming, and anaesthetists have been at the forefront of mitigating their impact. The NHS reports a reduction in emissions from volatile anaesthetic gases from 8000 t CO_2_e in 2018 to 1000 t CO_2_e in 2025 [39]. This is largely due to the significant reduction in the use of desflurane, a volatile gas with a global warming potential (GWP) 2540 times greater than CO_2_ over 100 years [40]. The European Commission has proposed a ban on its use, which will commence in January 2026 [41].

Additionally, we are seeing large reductions in emissions from nitrous oxide in anaesthesia. This has been largely due to the decommissioning of piped networks, which have been shown to result in the loss of up to 99% nitrous oxide through leaks and expired cylinders [42].

## Procurement and supply chain reform

It is estimated that supply chains are responsible for over half of healthcare-related emissions globally [43]. Healthcare systems do not control these emissions directly; however, they exercise considerable purchasing power and can influence the organisations that do. NHS England has taken many steps to encourage suppliers to decarbonise their activities to help meet their target of Net Zero Supply Chain by 2030 [16]. These include mandating all providers of contracts over £ 5 million to publish a carbon reduction plan for Scope 1 and 2 emissions, with a requirement for all contracts by 2027 [44].

The complexity, decentralisation and globalisation of supply chains pose challenges to their decarbonisation. To tackle this, at COP26 the Sustainable Markets Initiative Health Systems Task Force was established, a public–private partnership that brings together CEOs from leading pharmaceutical companies and global healthcare providers [43]. Private sector members have committed to pledges such as Net Zero Emissions by 2045 and 80–100% renewable power for their own operations by 2030 [43].

An important tool for assessing the environmental consequences of disposable items is the LCA

An important tool for assessing the environmental impact of single-use versus re-usable products is a *life cycle assessment* (LCA), a methodology that aims to comprehensively assess the environmental impact of a product, process or service, conducted in-line with international standards [45]. These LCAs have been used to provide an evidence base for a shift from single-use instruments to re-usable ones, for example re-usable laryngoscopes [46] and vaginal speculums [47]. These highlight how the carbon hotspots of single-use devices often originate in raw material extraction and disposal (the ability to recycle/re-process could then have a large impact), and for re-usable devices the environmental impact is largely dependent on the energy used to reprocess devices, which can be mitigated with renewable sources [46].

## Conclusions

The health sector must reconcile its imperative to protect health with the reality that its current operations are detrimental to the health of people and the planet. Climate mitigation in healthcare is both a moral obligation and a practical necessity. Many sustainability interventions are also cost-effective *and *provide better-quality patient care.

Nonetheless, certain barriers do exist. Key challenges facing healthcare systems include financial constraints, lack of regulatory incentives, fragmented governance and resistance to change. Overcoming these obstacles requires a coordinated approach that engages key stakeholders such as governments, NGOs, industry partners, professionals and the public. It is also important to recognise that decarbonisation should not compromise patient care, or worsen health inequalities. To prevent this, intersectional analysis should be applied to proposed interventions as well as to assessments of the long-term effects of interventions on health.

Climate change is one of the most pressing threats to human health. By reducing carbon emissions and building climate-resilient health systems, healthcare can model transformation necessary across society.

## Practical conclusion

- Climate change is considered the greatest threat to human health.
- Paradoxically, the healthcare sector accounts for a significant proportion of greenhouse gas (GHG) emissions.
- The effects of climate change on health are unevenly distributed, thereby exacerbating inequalities in healthcare.
- Infrastructure accounts for a significant proportion of the overall impact of healthcare on the environment.
- The certification of green buildings helps to accelerate the adoption of key technologies in green building design.
- Plant-based meals in hospitals reduce the environmental impact of food and have a positive effect on patient health.
- Focusing on the prevention of health impairments is inherently sustainable, as it reduces the need for healthcare services and their use.
- An important tool for assessing the environmental impact of disposable items is life cycle assessment (LCA).

## Declarations

For this article no studies with human participants or animals were performed by any of the authors. All studies mentioned were in accordance with the ethical standards indicated in each case.

The supplement containing this article is not sponsored by industry.
